# Twin Pregnancies, Crown-rump Length and Birthweight Discordancy: The Influence of Chorionicity

**DOI:** 10.1055/s-0040-1712128

**Published:** 2020-06-19

**Authors:** Joana Sousa Nunes, Mário Sousa, Nuno Montenegro, Alexandra Matias

**Affiliations:** 1 Obstetrics & Gynecology Department, Senhora da Oliveira Hospital, Guimarães, Portugal; 2 Obstetrics & Gynecology Department, Faculty of Medicine, Minho University, Braga, Portugal; 3 Laboratory of Cell Biology, Microscopy Department, Porto Hospital Centre, Institute of Biomedical Sciences Abel Salazar, Porto University, Porto, Portugal; 4 Obstetrics & Gynecology Department, São João Hospital & University Centre, Porto, Portugal

**Keywords:** fetal growth, growth discordancy, crown-rump length, birthweight, chorionicity, twins, crescimento fetal, discordância de crescimento, comprimento craniocaudal, peso ao nascimento, corionicidade, gêmeos

## Abstract

**Objective** The purpose of the present study was to analyze the influence of chorionicity in the biometric parameters crown-rump length (CRL), birthweight (BW), crown-rump length discordancy (CRLD) and birthweight discordancy (BWD), determine the correlation between these latter two in cases of intertwin discordancy, and to analyze the influence of chronicity in the presence of these discordancies with clinical relevance (> 10% and > 15%, respectively).

**Methods** The present study was a retrospective study based on the twin pregnancy database of the Centro Hospitalar S. João (2010–2015), including 486 fetuses among 66 monochorionic (MC) and 177 dichorionic gestations (DC). The inclusion criteria were multiple pregnancies with 2 fetuses and healthy twin gestations. The exclusion criteria were trichorionic gestations and pregnancies with inconclusive chorionicity, multiple pregnancy with ≥ 3 fetuses and pathological twin gestations.

**Results** No statistically significant difference was found in BW ( *p* = 0.09) and in its discordancy *(p =* 0.06) nor in CRL *(p =* 0.48) and its discordancy *(p =* 0.74) between MCs and DCs. Crown-rump length discordancy and birthweight discordancy were correlated by the regression line “BWD = 0.8864 x CRLD + 0.0743,” with r ^2^ = 0.1599. Crown-rump length discordancy > 10% was found in 7.58% of monochorionic and in 13.56% of dichorionic twins. Birthweight discordancy > 15% was detected in 16.67% of monochorionic and in 31.64% of dichorionic twins.

**Conclusion** No statistically significant influence of chorionicity was identified in both birthweight and birthweight discordancy, as in crown-rump length and crown-rump length discordancy. Birthweight discordancy was correlated to crown-rump length discordancy in 20% of cases.

## Resumo

**Objetivo** O objetivo do presente estudo foi analisar a influência da corionicidade nos parâmetros biométricos comprimento craniocaudal, peso ao nascimento, discordância de comprimento craniocaudal e discordância de peso ao nascimento, determinar a correlação entre estes dois últimos caso haja discordância intergemelar e analisar a influência da corionicidade na presença destas discordâncias com relevância clínica (> 10% e > 15%, respectivamente).

**Métodos** O presente estudo foi um estudo retrospectivo baseado na base de dados de gestações gemelares do Centro Hospitalar S. João (2010–2015), incluindo 486 fetos de 66 gestações monocoriônicas e 177 dicoriônicas. Os critérios de inclusão foram gestações múltiplas de 2 fetos e gestações gemelares saudáveis. Os critérios de exclusão foram gestações tricoriônicas ou de corionicidade inconclusiva, gestações múltiplas com ≥ 3 fetos e gestações gemelares patológicas.

**Resultados** Não se encontrou diferença estatisticamente significativa no peso ao nascimento ( *p* = 0,09) e sua discordância ( *p* = 0,06) nem no comprimento craniocaudal ( *p* = 0,48) e sua discordância ( *p* = 0,74) entre gestações monocoriônicas e dicoriônicas. Considerando todas as gestações, as discordâncias de comprimento craniocaudal e peso ao nascimento foram correlacionadas pela reta de regressão “discordância de peso ao nascimento = 0.8864 x discordância de comprimento craniocaudal + 0.0743,” com r ^2^ = 0,1599. A discordância de comprimento craniocaudal > 10% descobriu-se em 7.58% das gestações monocoriônicas e em 13.56% das dicoriônicas. A discordância de peso ao nascimento > 15% detectou-se em 16.67% das gestações monocoriônicas e em 31.64% das dicoriônicas.

**Conclusão** Não se identificou influência estatisticamente significativa no peso ao nascimento e sua discordância, bem como no comprimento craniocaudal e sua discordância. A discordância de peso ao nascimento correlacionou-se com a discordância de comprimento craniocaudal em 20% dos casos.

## Introduction

Twinning is increasing worldwide with increased maternal age and more common use of assisted reproduction. The higher risk of mortality and morbidity in multiples is widely recognized. 1 2 3 5 6 7 10 11 12 16 17 18 19 24 26 30 32 34 35 According to the classification of twin pregnancies, no matter how many fetuses we are dealing with (zygosity), what really counts for defining perinatal outcome of twin pregnancies is the type of placentation (chorionicity). 1 2 4 5 6 10 23 35 However, in the literature, other authors favor a contrasting opinion. 7 Furthermore, the importance of chorionicity on twin growth patterns is well-established, being monochorionic twin gestations (MC) the ones with a less favorable scenario. In fact, growth restriction, low birthweight (BW) and birthweight discordancy > 25% are common findings in multiple pregnancies, mainly among MC twins. 8 21 23 24 27 28 29 30 31 35 36 Birthweight discordancy affects up to 20% of MC and only 8% of dichorionic twin gestations (DC), being unequal placental sharing the major contributor. 21 28 This condition can be divided into 3 categories: < 15% (concordant growth), 15–25% (mildly discordant growth) and > 25% (severely discordant growth). 20 22 24 25 26 30 36 These abnormal growth patterns related to chorionicity cause worse outcomes since the obstetric management is not well-established yet. 28 31 The use of first trimester transvaginal ultrasonography is therefore mandatory to obtain an early accurate determination of multiple gestations, to define their chorionicity and zygosity, as well as to calculate some important biometric parameters such as crown-rump length (CRL) and its inter-twin discordancy. 1 9 35 Some authors have analyzed this inter-twin CRL discordancy (CRLD), which is considered to be of major clinical importance when ≥ 10%, as a predictor of an increased risk for fetal anomalies and growth restriction, affecting BW in the long run. 32 33 Contrarily, other studies classified the CRLD as a poor predictor of adverse outcome due to its lack of accuracy, proving useless as a screening method in the current clinical practice. 33 34 The purpose of the present study was to analyze the influence of chorionicity in the biometric parameters CRL, BW, CRLD and BWD, determine the correlation between these latter two in cases of inter-twin discordancy, and to analyze the influence of chronicity in the presence of these discordancies with clinical relevance (> 10% and > 15%, respectively).

## Methods

The present study was a retrospective study based on the twin pregnancy database of Centro Hospitalar S. João related to a period of 5 years (2010–2015). We considered a total of 706 fetuses. From those, we included 486 fetuses, 132 from 66 MC (each one with fetus 1 and fetus 2) and 354 from 177 DC (each one with fetus 1 and fetus 2). The inclusion criteria were multiple pregnancies with 2 fetuses and healthy twin gestations. The exclusion criteria were trichorionic gestations and pregnancies with inconclusive chorionicity, multiple pregnancies with ≥ 3 fetuses and pathological twin gestations. By healthy and nonpathological twin gestations, the authors considered gestations without malformed fetuses or other fetal pathologies that could interfere in the spontaneous inter-twin discordancy, congenital anomalies, twin-to-twin transfusion syndrome, selective intrauterine growth restriction and presence of maternal pathologies (pre-eclampsia, diabetes, etc). In this database, we considered two biometric parameters: CRL, evaluated in the 1st trimester obstetric ultrasound (performed between the 11 ^th^ and 14 ^th^ weeks of gestation), as well as BW, confirmed after birth. Chorionicity was confirmed in the 1 ^st^ trimester obstetric ultrasound. The defined objectives for the statistical analysis were: 1 ^st^ – analyze individually the biometric parameters CRL, BW, CRLD and birthweight discordancy, according to chorionicity (among 3 different samples – all fetuses, only fetuses 1 and only fetuses 2–concerning CRL and BW, and among all gestations, concerning CRLD and birthweight discordancy); 2 ^nd^ - determine the association between CRLD and birthweight discordancy and analyze the regression line of their association graph; 3 ^rd^ – discordancy of CRL and discordancy of BW were analyzed for both MCs and DCs considering as clinically relevant a CRLD > 10% and a birthweight discordancy > 15%. The discordancy of each parameter was calculated by using the ratio between the difference of the measurements of the two fetuses of the same gestation and the larger measurement between them. The first objective was used to demonstrate that the population of MCs and DCs is comparable since the study included only twin pregnancies that had a normal outcome. In this case, it is possible to evaluate the early ultrasound parameters and their birthweight discordancy in the two populations studied. The statistical analysis was performed using IBM SPSS Statistics for Windows, Version 23 (IBM Corp., Armonk, NY, USA), and the chosen significance value for the applied statistical tests was 0.05. The present investigation was approved by the ethics committee of the hospital and authorized by the Centro Hospitalar S. João Board of Directors.

## Results

For the 1 ^st^ objective, we analyzed the data from 3 different samples (all fetuses, fetuses 1 and fetuses 2) concerning CRL and BW according to their chorionicity. Regarding the influence of chorionicity type among all fetuses, we obtained, with the parametric *t* -test, *p* = 0.48 (> 0.05), for CRL, and *p* = 0.09 (> 0.05), for BW. Concerning the influence of chorionicity for fetuses 1, we obtained, with the parametric *t* -test, *p* = 0.68 (> 0.05), for the CRL, and *p* = 0.12 (> 0.05), for BW. In what concerns the influence of chorionicity for fetuses 2, we obtained, with the parametric *t* -test, *p* = 0.56 (> 0.05), for CRL, and *p* = 0.40 (> 0.05), for BW. All these results are depicted in Table 1 .

**Table 1:** Influence of chorionicity in crown-rump length (mm) and birthweight (g)

	**Crown-rump length (mm)**			
	**Total**	**Monochorionic**	**Dichorionic**	***p-value***
All fetuses	61.32 ± 11.27	62.01 ± 14.12	61.07 ± 10.03	0.48
Fetuses 1	61.35 ± 11.27	62.08 ± 13.76	61.31 ± 9.92	0.68
Fetuses 2	61.34 ± 11.28	61.94 ± 14.59	60.82 ± 10.17	0.56
	**Birthweight (g)**			
	**Total**	**Monochorionic**	**Dichorionic**	***p-value***
All fetuses	2219.07 ± 542.21	2150.87 ± 554.75	2244.50 ± 536.81	0.09
Fetuses 1	2220.12 ± 542.27	2168.26 ± 591.43	2290.17 ± 522.44	0.12
Fetuses 2	2218.69 ± 542.70	2133.48 ± 519.44	2198.84 ± 548.47	0.40

Second, we also analyzed, among all gestations, the influence of chorionicity in CRLD and birthweight discordancy, obtaining *p* = 0.74 (> 0.05) and *p* = 0.06 (> 0.05), respectively. These results are displayed in Table 2 .

**Table 2:** Influence of chorionicity type in crown-rump length discordancy (CRLD) and in birthweight discordancy (birthweight discordancy) (%)

	Crown-rump length discordancy (CRDL) (%)			
	Total	Monochorionic	Dichorionic	*P*
All gestations	5,00 ± 4,80	4,90 ± 5,00	5,10 ± 4,70	0,74
	Birthweight discordancy (birthweight discordancy) (%)			
	Total	Monochorionic	Dichorionic	*P*
All gestations	12.00 ± 10,65	9.80 ± 8,80	12,70 ± 11,20	0,06

**Fig. 1:**
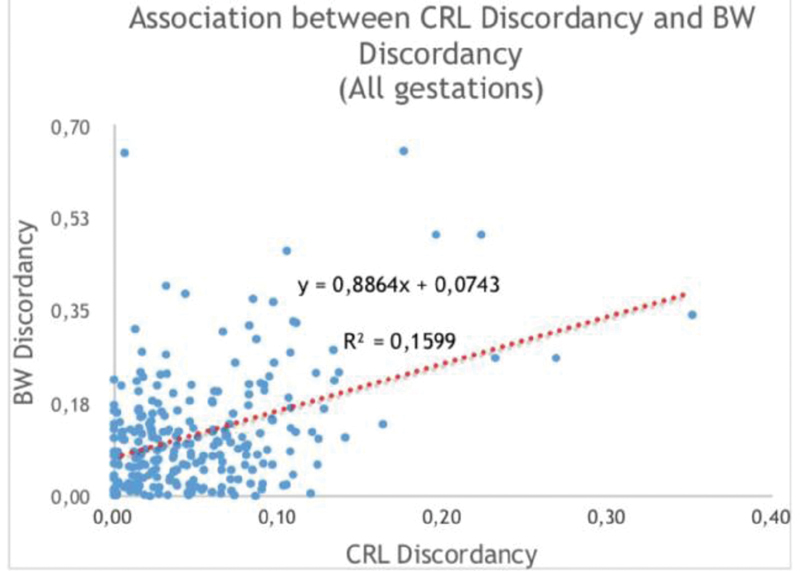
Correlation between crown-rump length discordancy (CRLD) and birthweight discordancy (birthweight discordancy), among all gestations.

Concerning the 2 ^nd^ objective, we determined the association between CRLD and birthweight discordancy and analyzed the regression line of their association graph. Among all gestations, the correlation between CRLD and birthweight discordancy can be seen in Fig. 1 , in which the regression line is defined by birthweight discordancy = 0.8864 x CRLD + 0.0743, with r2 = 0.1599, being r2 (coefficient of determination) the variation of birthweight discordancy explained by CRLD.

**Fig. 2:**
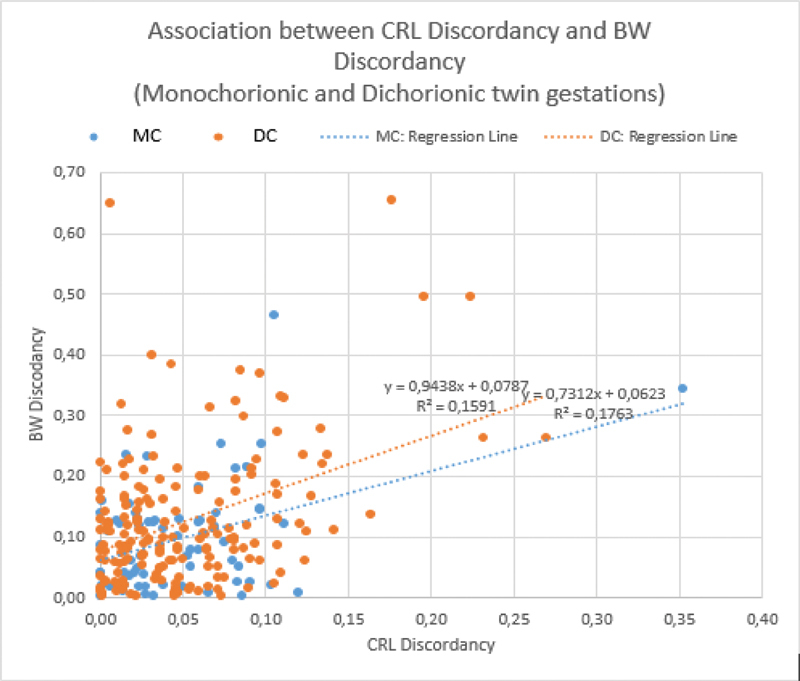
Correlation between crown-rump length discordancy (CRLD) and birthweight discordancy (birthweight discordancy), among monochorionic and dichorionic gestations.

The same analysis was performed among MCs and DCs ( Fig. 2 ). In MCs, the association graph had a regression line defined by birthweight discordancy = 0.7312 x CRLD + 0.0623, with r2 = 0.1763; and, in DCs, the association graph had a regression line defined by birthweight discordancy = 0.9438 x CRLD + 0,0787, with r2 = 0.1591, similar to the results showed among all gestations.

Concerning the 3rd objective, CRL discordancy and BW discordancy for both MC and DC were analyzed. According to the literature, discordancy in CRL ≥ 10% and, in BW, ≥ 15% was considered of major clinical importance. The results achieved showed that 7.58% of MCs showed a CRLD of at least 10%, against 13.56% among DCs. It was also verified that 16.67% of MCs had a birthweight discordancy of at least 15%, against 31.64% among DCs.

## Discussion

No statistically significant differences for CRL and BW according to chorionicity were found, but a borderline, although non-statistically significant difference, was observed for BW. A similar situation was identified for the influence of chorionicity in birthweight discordancy and CRLD. This can be explained by an early developmental phase in which the CRL measurement is performed, and therefore the influence of chorionicity in fetal growth may not be noticeable until later in pregnancy when the BW is estimated. The fetal growth progression and the later phases of development will possibly allow for more biometric differences and diverse growth of the two fetuses when there are two placentas available (DCs). This first conclusion was compatible with the results found in the reviewed literature. 1 2 3 4 5 6 10 23 Maybe in future studies with a larger sample, this influence of chorionicity in BW and birthweight discordancy will become more apparent.

Among all gestations, nearly 16% of the birthweight discordancy is correlated to CRLD. Among MCs, nearly 18% of the birthweight discordancy is correlated to the CRLD, not very different from what happens in DCs, in which nearly 16% of the BWD is correlated to the CRLD. BWD may be correlated in this present extension to CRLD, but not really explained by it since other variables were not studied. So, it would be interesting in future studies to clarify the other putative determinants that could explain ∼ 80% of the birthweight discordancy other than CRLD, which only seems to account for nearly 20%. This second conclusion was matched with the results found by other authors, such as Grande et al. 32

There is a higher percentage of discordancy in CRL ≥ 10% in DCs (13.56%) than in MCs (7.58%). Regarding the BW discordancy, there is also a greater percentage of major and clinically relevant discordancy in DCs (31.54%) than in MCs (16.67%). This third conclusion was contradicted by the reviewed literature, being necessary some other studies to clear up this point. 28 Moreover, pathological cases with selective intrauterine growth restriction were one of the exclusion criteria of the present study, and this is probably one of the reasons why birthweight discordancy is greater in dichorionic pregnancies. Therefore, care should be taken in the generalization of this conclusion by the analysis of the data collected.

The present study had some possible limitations, such as the intraobserver and interobserver variability in the measurements of the biometric parameters, since CRL and BW measurements were performed by different certified health professionals, as well as the limitation related to the sample length, which should be larger in future studies to clarify the influence of chorionicity in later stages of twin pregnancies. Moreover, the statistical analysis would be more interesting if applied in the future in a prospective study, accompanying the twin pregnancies until the birth of the babies or even trying to go forward perceiving the later consequences of the birthweight discordancy.

As strengths, the present study raises the issue of the possible influence of chorionicity in twins' growth and the future consequences of birthweight discordancy and CRLD in the potential of fetal growth according to the type of placentation.

## Conclusion

According to the main objectives of the present study, no statistically significant influence of chorionicity could be identified in both BW and birthweight discordancy, as well as in CRL and CRLD. Nevertheless, birthweight discordancy was explained in nearly 20% by the influence of CRLD. This findings should let all the health providers aware of the main importance of strict and precocious twin pregnancies' surveillance to prevent any disturbance of fetal growth and development.
